# ONE HUNDRED MILLION HEALTHIER LIVES: MEASURING WHAT MATTERS

**DOI:** 10.1093/geroni/igz038.2931

**Published:** 2019-11-08

**Authors:** Kathleen A Cameron, Angelica Herrera-Venson

**Affiliations:** National Council on Aging, Arlington, Virginia, United States

## Abstract

The rise of chronic disease, an aging population, inequity in health care access and outcomes, and new technology demand a fundamentally different approach to fostering health—one that brings sectors together to address the social and behavioral determinants of health. To address these concerns, NCOA is leading the Aging Hub of the 100 Million Healthier Lives (100MLives), an initiative convened by the Institute for Healthcare Improvement. 100MLives is a collaboration of change agents across sectors working to transform how the world thinks and acts to improve health, well-being, and equity across the lifespan. NCOA is collaborating with national and local organizations, as well as federal agencies to support a nationwide ecosystem for measuring, fostering, and bringing to scale innovations that address social and behavioral determinants of health. The Aging Hub is committed to consistently measuring what matters (adult well-being assessment-AWA), learn what programs and services within the aging network improve a person’s life, and work with partners to scale those programs. The initiative encourages use of the Adult Well-Being Assessment, a validated 7-question survey that asks individuals to rate their overall well-being; financial, physical, mental, and spiritual health; and social support. Shared use of these outcome measures can make it easier for the aging network to demonstrate the impact of their programs and services. Join NCOA to learn about progress to date, including a report on AWA and demographic data collected by the Baltimore County Department with over 10,000 older adults and how data is being used to improve service delivery.

